# Automatic multi-vessel volume flow calculation with 4D flow CMR

**DOI:** 10.1186/1532-429X-17-S1-O45

**Published:** 2015-02-03

**Authors:** Mariana Bustamante, Petter Dyverfeldt, Sven Petersson, Jonatan Eriksson, Carl Johan Carlhall, Tino Ebbers

**Affiliations:** 1 Medical and Health Sciences, Linköping University, Linköping, Sweden

## Background

Volume flow analysis is essential in the assessment of many cardiovascular diseases such as valvular regurgitation, intra-cardiac shunt, and complex congenital heart diseases.

Clinically, CMR-based volume flow analysis is performed using 2D flow CMR. This requires user-dependent and time-consuming positioning of 2D planes in each vessel while the patient is still in the scanner.

Previous studies have demonstrated that 4D flow CMR permits accurate volume flow assessment. However, retrospective plane-positioning and region-of-interest delineation requires time-consuming user interaction.

The aim of this study was to develop an automatic method for volume flow analysis in the great thoracic vessels using 4D flow CMR.

## Methods

**Figure 1:**
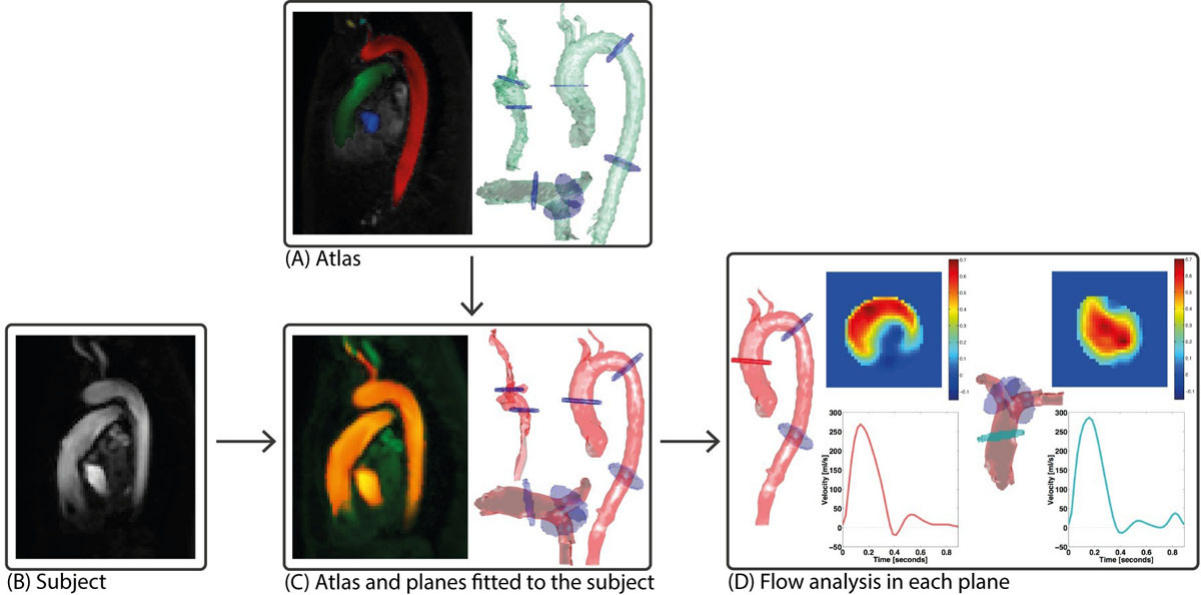
Schematic illustration of the analysis method. (A) An atlas was created from one healthy volunteer's PC-MRA, with planes positioned in locations of interest. (B) A PC-MRA is created from another subject's 4D flow CMR received as input. (C) The images are registered in order to fit the vessels and planes from the atlas to the input subject. (D) Flow analysis is performed in each plane.

The automatic multi-vessel volume flow calculation method is illustrated in Figure 1. An atlas (reference vessel segmentation) was created by manual segmentation of the great thoracic vessels in one healthy volunteer. The segmentation was done on a 3D PC-MRA which was derived from the 4D flow CMR data. Analysis planes for volume flow determination were positioned in the proximal ascending aorta and pulmonary trunk.

For each subject, the atlas' PC-MRA was registered to the subject's PC-MRA. In this way, the atlas' vessels and analysis planes were transformed into the subject's vessels. The transformed atlas was transferred to all timeframes using the 4D flow CMR magnitude image, resulting in a time-resolved segmentation that follows the motion of the vessels over the cardiac cycle.

Finally, the volume flow was automatically calculated for each plane using the time-resolved atlas as a mask to account for vessel location, shape and movement.

The method was evaluated in a group of subjects composed of 10 healthy volunteers and 11 patients with heart failure of different etiologies. Results in the proximal ascending aorta were compared against volume flow values obtained by manual segmentation. Additionally, the pulmonary-to-aortic flow ratio (Qp/Qs) was assessed.

## Results

Volume flow values were successfully obtained in 20 out of 21 subjects. In the failed case, the subject's cardiovascular morphology differed significantly from the atlas' morphology.

**Figure 2:**
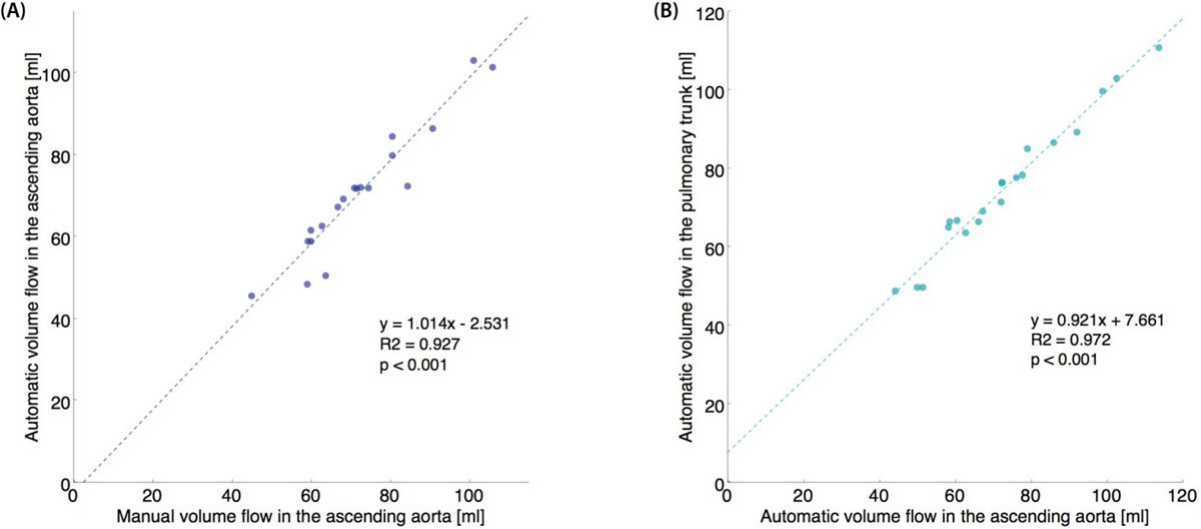
Regression analysis of (A) manual vs. automatic volume flows in the proximal ascending aorta, and (B) automatically measured volume flows in the pulmonary trunk vs. proximal ascending aorta (Qp/Qs). Both comparisons yielded very strong correlation: R2 = 0.927 and R2 = 0.972, respectively (p < 0.001 in both cases).

A very strong correlation between the automatically and manually obtained volume flow values in the ascending aorta was found (Figure 2a). The automatic volume flow values obtained in the proximal ascending aorta and the pulmonary trunk also showed very strong correlation (Figure 2b).

## Conclusions

A method for automatic volume flow analysis of 4D flow CMR data was developed and evaluated. This method permits automatic analysis of flow in any number of planes located in the major vessels of a subject. Future work includes evaluation of the method in patients with more severe cardiovascular abnormalities, such as complex congenital heart disease, which may require a stronger registration method or the creation of disease-specific atlases. Also, other interesting plane locations will be evaluated.

## Funding

This study is founded by the European Research Council, the Swedish Research Council and the Swedish Heart-Lung Foundation.

